# Correction: Structural modification of olibergin A, an isoflavonoid, from *Dalbergia stipulacea* Roxb. and its cytotoxicity

**DOI:** 10.1039/d2ra90067j

**Published:** 2022-07-07

**Authors:** Supakorn Arthan, Priyapan Posri, Sookkawath Walunchapruk, Thanaset Senawong, Chavi Yenjai

**Affiliations:** Department of Chemistry and Center of Excellence for Innovation in Chemistry, Faculty of Science, Natural Products Research Unit, Khon Kaen University Khon Kaen 40002 Thailand chayen@kku.ac.th +66-043-009700, ext. 42174 Tel: +66-043-009700 ext. 42175; Department of Biochemistry, Faculty of Science, Natural Products Research Unit, Khon Kaen University Khon Kaen 40002 Thailand

## Abstract

Correction for ‘Structural modification of olibergin A, an isoflavonoid, from *Dalbergia stipulacea* Roxb. and its cytotoxicity’ by Supakorn Arthan *et al.*, *RSC Adv.*, 2022, **12**, 17837–17845, https://doi.org/10.1039/D2RA02865D.

The authors regret that incorrect IUPAC nomenclature was used for compound **4** in the manuscript. The correct nomenclature for compound **4** is “5-hydroxy-2′,5′-dimethoxyisoflavone 7,4′-dipentanoate”.

In addition, for compound **3**, the spectroscopic data contains the phrase “cmb:b:line” for the two signals at 168.8 and 168 ppm. The correct spectroscopic data for compound **3** is “168.8 (MeCOO-7), 168.0 (MeCOO-4′)”.

The Royal Society of Chemistry apologises for these errors and any consequent inconvenience to authors and readers.

## Supplementary Material

